# Immunization with advanced glycation end products modified low density lipoprotein inhibits atherosclerosis progression in diabetic apoE and LDLR null mice

**DOI:** 10.1186/s12933-014-0151-6

**Published:** 2014-11-13

**Authors:** Lin Zhu, Zhiqing He, Feng Wu, Ru Ding, Qixia Jiang, Jiayou Zhang, Min Fan, Xing Wang, Bengtsson Eva, Nilsson Jan, Chun Liang, Zonggui Wu

**Affiliations:** Department of Cardiology, Shanghai Changzheng Hospital, Second Military Medical University, No. 415 Fengyang Road, Shanghai, 200003 People’s Republic of China; 457th hospital of PLA, Wuhan, People’s Republic of China; Department of Research, Center for Stem Cell Biology, Roger Williams Medical Center, Boston University School of Medicine, Providence, RI USA; Experimental Cardiovascular Research, CRC 91:12, Lund University, Entrance 72, Skåne University Hospital Malmö, SE-205 02 Malmö, Sweden

**Keywords:** Immunization, Advanced glycation end products, Low density lipoprotein, Atherosclerosis, Diabetes

## Abstract

**Background:**

Diabetes accelerates atherosclerosis through undefined molecular mechanisms. Hyperglycemia induces formation of advanced glycation end product (AGE)-modified low-density lipoprotein (LDL). Anti-AGE-LDL autoantibodies favor atherosclerosis (AS) progression in humans, while anti oxidized LDL immunization inhibits AS in hypercholesterolemic, non-diabetic mice. We here investigated if AGE-LDL immunization protects against AS in diabetic mice.

**Methods:**

After diabetes induction with streptozotocin and high fat diet, both low density lipoprotein receptor (LDLR)−/− and apoE female mice were randomized to: AGE-LDL immunization with aluminum hydroxide (Alum) adjuvant; Alum alone; or PBS.

**Results:**

AGE-LDL immunization: significantly reduced AS; induced specific plasma IgM and IgG antibodies; upregulated splenic Th2, Treg and IL-10 levels, without altering Th1 or Th17 cells; and increased serum high density lipoprotein(HDL) while numerically lowering HbA1c levels.

**Conclusions:**

Subcutaneous immunization with AGE-LDL significantly inhibits atherosclerosis progression in hyperlipidemic diabetic mice possibly through activation of specific humoral and cell mediated immune responses and metabolic control improvement.

**Electronic supplementary material:**

The online version of this article (doi:10.1186/s12933-014-0151-6) contains supplementary material, which is available to authorized users.

## Introduction

Diabetes mellitus through as yet undefined mechanisms worsens short and long term clinical prognosis of cardiovascular disease (CVD) manifested as asymptomatic ischemic events and increased morbidity and mortality [1]. No current therapy specifically targets diabetes-induced CVD, however, promising immunization strategies against AS have been developed [2-9], supported by: association between autoantibodies against oxidized plasma low density lipoprotein (ox-LDL) and AS in humans [10]; reduced AS lesion formation in mice after ox-LDL immunization [11]; and AS regression induction in Apobec-1−/− /LDL receptor (LDLR) null mice by recombinant antibodies against aldehyde-modified apoB100 peptide 661–680 [12]. Diabetes is associated with increased protein glycation and generation of AGE; in particular, AGE-LDL appears suitable as basis for a vaccine against AS in diabetes because it accelerates AS progression via different signaling pathways [13-21], and similar to ox-LDL, immune complexes containing anti-AGE-LDL autoantibodies in diabetics are associated with AS [22,23]. In this study, we immunized diabetic apoE−/− and LDLR−/− mice with AGE-LDL to assess ability of this strategy to reduce AS.

## Material and methods

### LDL isolation and glycation

LDL was isolated from pooled plasma of healthy volunteers by ultracentrifugation. Glycated LDL was prepared by incubation of LDL with 0.2 M glucose for 4 weeks at 37°C with antioxidants (1 mg/ml EDTA and 1 μM butylated hydroxytoluene) under sterile conditions, and extensively dialyzed before use. Oxidized LDL (ox-LDL) was prepared by incubating native LDL (nLDL) under sterile conditions with 5 μM CuCl_2_ without antioxidant protection for 3 hours as previously described [24]. The oxidative reaction was stopped by addition of EDTA, dialyzed extensively against PBS under sterile conditions, stored at 4°C, and used within 7 days. Protein glycation was measured by the thiobarbituric assay (TBA) and expressed as nmoles of 5-hydroxymethylfurfural (HMF) per mg protein [25] and by trinitrobenzene sulphonic acid assay (TNBSA) [26]. Protein concentration was measured by a modified Lowry method as previously reported [27]. LDL oxidation was assayed by measuring the thiobarbituric acid reactive substances (TBARS) and carbonyls [28]. The electric charge of LDLs (including nLDL, AGE-LDL and ox-LDL) were evaluated by agarose gel electrophoresis as previously reported [29].

### Ethics statement

The study had been reviewed by the ethics committee of Second Military Medical University, and performed in accordance with the ethical standards as per the 1964 Declaration of Helsinki. Informed consent was obtained from all volunteers of plasma collection.

### Mice, immunization, and tissue preparation

Local Animal Care and Use Committee at Second Military Medical University approved the entire experimental protocol used in the study. All surgery was performed under anesthesia, and all efforts were made to minimize suffering in compliance with the ARRIVE guidelines on animal research [30].

Female apoE−/− and LDLR−/− mice on a C57BL/6 background from Jackson Laboratory (USA) were used (n = 36, 3 groups of 12 apoE null mice and n = 30, 3 groups of 10 LDLR null mice). From 6 weeks of age mice were fed ad libitum a high-cholesterol diet (0.15% cholesterol, 21% fat); made diabetic with intraperitoneal injections of streptozotocin according to a modified protocol (50 mg/kg per day dissolved in 200 μl citrate buffer, pH 7.4 for 7 days) [31]; and monitored for blood glucose and HbA1c once weekly until euthanized. At 9 weeks of age the mice were injected subcutaneously (first immunization) in the dorsal area between scapulas, followed by a booster at 11, 13 and 15 weeks of age (Additional file 1: Figure S1). Aluminum hydroxide (Alum) (Biosecter, Denmark) was used as adjuvant and mixed with AGE-LDL (25 μg/dose) with 1:1 ratio in volume. The mixture with Alum was freshly prepared prior to each immunization. Alum alone or citrate buffer was used as controls.

All mice were euthanized at 24 weeks of age by exsanguination through cardiac puncture under anesthesia with pentobarbital sodium administered intraperitoneally. Spleens were immediately collected and immersed in prepared cold PBS containing antibiotics (Life, USA) for flow cytometry and real time PCR analysis without perfusion. After whole-body perfusion with phosphate-buffered saline (PBS) followed by Histochoice (Amresco), the heart and the aortic arch were dissected out and stored in Histochoice at 4°C until processing. The aorta was dissected free of external fat and connective tissue, cut longitudinally, and mounted *en face*, lumen side up, on polylysine and 3-aminopropyltriethoxysilane (APES)-coated slides (Bolster, Wuhan).

### Aorta staining

*En face* preparations of the aorta were washed in distilled water, dipped in 78% methanol, and stained for 40 minutes in 0.16% Oil-Red-O solution as previously described [32]. The cover slides were mounted with a water-soluble mounting media. Lipids are stained red. The slides were scanned and digitized with Epson 4990, and stained plaque areas in total aorta were quantified blindly by computer aided morphometry software (Image J).

### Analysis of plaques

Staining and quantification of plaque area in aorta and subvalvular plaque macrophage content were done as previously described [32]. The aortic arch was embedded in OCT (Tissue-Tek, Sakura, Japan) and 10-μm frozen sections were collected. The sections were dipped briefly in 60% isopropanol and stained in 0.24% Oil Red-O in 60% isopropanol for 20 minutes. Sections were briefly washed in 60% isopropanol, then washed in water and counter-stained with hematoxylin. Plaque collagen content was assessed with Masson’s trichrome staining kits (Maximbio, Fuzhou, China). Slides used for staining with rat anti-mouse MOMA-2 (monocyte/macrophage, MAB1852, Millipore USA) and alpha-smooth muscle actin antibodies (A5228, Sigma, USA) diluted in 10% rat serum in PBS incubated at 4°C overnight, were first fixed in ice-cold acetone for 10 minutes, washed in PBS for 5 minutes, and then blocked with 10% mouse serum in PBS for 30 minutes and quickly dipped in PBS. Biotinylated rabbit anti-rat IgG was used as secondary antibody and DAB detection kit for color development (Maximbio, Fuzhou, China). Omissions of the primary antibodies were used as negative control [33]. Stained area was quantified blindly by computer aided morphometry software (Image J).

### Serum analyses

Blood samples were collected by either tail-vein nick (glucose) or cardiac puncture (HbA1c, lipid and cytokine analysis). Non-fasting glucose levels were measured using an Accu-Chek Compact Meter (Roche Diagnostics, Indianapolis, IN) once a week. HbA1c levels were measured using A1cNow + Monitors (Metrika, Sunnyvale, CA) [34]. Total plasma cholesterol was quantified by colorimetric assays (Infinity Total Cholesterol Reagent; Sigma). Plasma high density lipoprotein cholesterol (HDL-C) was determined by precipitating non–HDL-C (Wako Diagnostic) and then assaying the remaining cholesterol with the Infinity Total Cholesterol Reagent. Low density lipoprotein cholesterol (LDL-C) was determined by L-type LDL-C kit from Wako Chemicals (Richmond, Virginia, USA). IL-10, TGF-**β** and IFN-**γ** in serum and cytokines in splenocyte supernatants were measured with Milliplex kits (Millipore, USA) in a Luminex Multiplexing Instrument according to manufacturer’s instructions.

### Antibody assays

AGE-modified LDL were used for coating (200 μg/mL of each in PBS pH 7.4) microtiter plates (Nunc MaxiSorp, Denmark) in an overnight incubation at 4°C. Coated plates were washed with PBS with 0.05% Tween-20 and thereafter blocked with SuperBlock in Tris-buffered saline for 5 minutes at room temperature followed by an incubation of mouse serum diluted 1:50 in TBS-0.05% Tween-20 for 2 hours at room temperature and overnight at 4°C. After washing, bound antibodies were detected by using biotinylated goat anti-mouse IgM (AP500B, Millipore, USA) or IgG (B7264, Sigma, USA) or rat anti-mouse IgG1 or IgG2a secondary antibodies (RMG115 and RMG2a15, Millipore, USA) that were incubated for 2 hours at room temperature. The plates were washed, and bound biotinylated antibodies were detected by alkaline phosphatase–conjugated streptavidin (V559C, Promega, USA). The color reaction was developed using phosphatase substrate kit (Pierce). Absorbance at 405 nm was measured after 1 hour incubation at room temperature. Mean values were calculated after subtraction of background absorbance (n = 4 per mouse).

### Flow cytometry and intracellular cytokine staining

Flow cytometry was performed on a FACS Calibur (BD, USA) after staining with appropriate antibodies; data were analyzed using Flowjo software (USA). Primary labeled antibodies used were from BD Biosciences (anti-CD4) or from eBioscience (anti-FoxP3). To characterize the cytokine expression profiles of splenic CD4+ T cells from different vaccinated mice, cell suspensions were prepared as described before and evaluated by intracellular cytokine staining and FACS analysis. Briefly, splenocytes were prepared by mechanical disruption followed by incubation in erythrocyte lysis buffer (Qiagen, USA) and extensively washed. 2 × 10^6^ spleen were stimulated for 4 h at 37°C in 7.5% CO_2_ with PMA (phorbol 12-myristate 13-acetate; 50 ng/ml, Sigma), ionomycin (500 ng/ml; Sigma) and monensin (2 ng/ml; Biolegend). All cells were incubated with Fc**γ**R block (BD Bioscience) followed by surface (anti-CD4, Biolegend) and then intracellular staining of IFN**γ** (eBioscience), IL-4, or IL-17 (Biolegend) and FoxP3 (eBioscience) according to manufacturer’s instructions. Lymphocytes were firstly gated using forward and side scatter plots, and then intracellular staining was performed within only CD4 positive T cells to characterize T-cell subtype (IFN**γ**^+^, IL-4^+^, or/and IL-17^+^, while FoxP3^+^ in CD4^+^CD25^+^). Fluorescence minus one (FMO) controls were stained in parallel using the panel of antibodies with sequential omission of one antibody, with the exception of the anti-Foxp3 antibody, which was replaced by an isotype control. One million cells, washed twice in PBS before fixation and permeabilization, were used for each test.

### Real-time polymerase chain reaction

RNA was isolated from spleens using the RNeasy kit from Qiagen (Hilden, Germany). Purity of obtained RNA was estimated by Nanodrop from Thermo (USA). RNA with an A260/A280 > 1.8 was used for cDNA synthesis. Reverse transcription was performed with PrimeScript™ 1st Strand cDNA Synthesis Kit (Takara, Japan) and cDNA amplified by iQ SYBR Green real-time PCR Supermix (Bio-rad, USA) using primers (Life Technology, USA) for FoxP3, IL-10, TGF-**β**, IFN-**γ** and Glyceraldehyde 3-phosphate dehydrogenase (GAPDH) in an CFX Connect real-time PCR detection system from Bio-rad. Data were analyzed based on relative expression method using the formula 2^-**ΔΔ**C^_T_, where **ΔΔ**C_T_ = **Δ**C_T_ (sample) – **Δ**C_T_ (calibrator = average CT values of all samples within each group), and **Δ**C_T_ is the C_T_ of the housekeeping gene (GAPDH) subtracted from the C_T_ of the target gene.

### Statistical analysis

Data are presented as mean ± standard deviation. Comparison among groups was performed using one-way ANOVA and Turkey post test using GraphPad Prism 5.01 (Graphpad software, La Jolla, CA, USA). Statistical significance was considered at the level p < 0.05.

## Results

### Characterization of AGE-LDL

Because in vivo glycation of LDL is very complex involving nonenzymatic reaction of glucose and its metabolites with the free amino groups of lysine [35], nLDL and ox-LDL were used as reference to characterize the AGE-LDL used as immunization agent. AGE-LDL showed extensive apoB glycation (TBA) and high lipid peroxide levels (TBARS assay); glycation was increased by >4-fold (7.35 ± 0.83 vs. 1.92 ± 1.30 nmoles HMF/mg protein) or >6-fold (1.82 ± 0.31 vs. 0.30 ± 0.21 nmoles MDA/mg protein, P < 0.05) relative to nLDL. TNBSA assays showed 29% reduction in free amino groups of apoB in AGE-LDL relative to n- or ox-LDL (0.32 ± 0.04 AU vs. 0.45 ± 0.08 AU or 0.40 ± 0.07 AU, respectively, P < 0.05) consistent with irreversible protein glycation (Table 1); while Rf measurement showed a 45% increase for AGE-LDL (0.74 ± 0.05, P < 0.05) and a 24% increase for ox-LDL (0.63 ± 0.08, P < 0.05) compared with nLDL (0.51 ± 0.05), indicating loss of positive charges due the glycation of Lys and Arg residues. In vitro generated AGE-LDL therefore had similar features to those of AGE-LDL collected from plasma in vivo and could be used as potential vaccination antigen.

**Table 1:** **Characterization of AGE-LDL**

	**nLDL**	**ox-LDL**	**AGE-LDL**
TBA (nmoles HMF/mg protein)	1.92 ± 1.30	4.41 ± 1.22*	7.35 ± 0.83**^#^
TNBSA (AU)	0.45 ± 0.08	0.40 ± 0.07*	0.32 ± 0.04*
TBARS (nmoles MDA/mg protein)	0.30 ± 0.21	4.17 ± 0.06**	1.82 ± 0.31*^#^
Rf (cm)	0.51 ± 0.05	0.63 ± 0.08*	0.74 ± 0.05*

*indicates p < 0.05 vs. nLDL, **indicates p < 0.01 vs. nLDL and ^#^indicates p < 0.05 vs. ox-LDL; nLDL: native low density lipoprotein; TBA: thiobarbituric assay; HMF: 5-hydroxymethylfurfural; TNBSA: trinitrobenzene sulphonic acid assay; TBARS: thiobarbituric acid reactive substances; MDA: malondialdehyde; Rf: relative electrophoretic mobility by agarose gel electrophoresis.

### Immunization with AGE-LDL inhibits atherosclerosis in murine diabetic models

**Figure 1:**
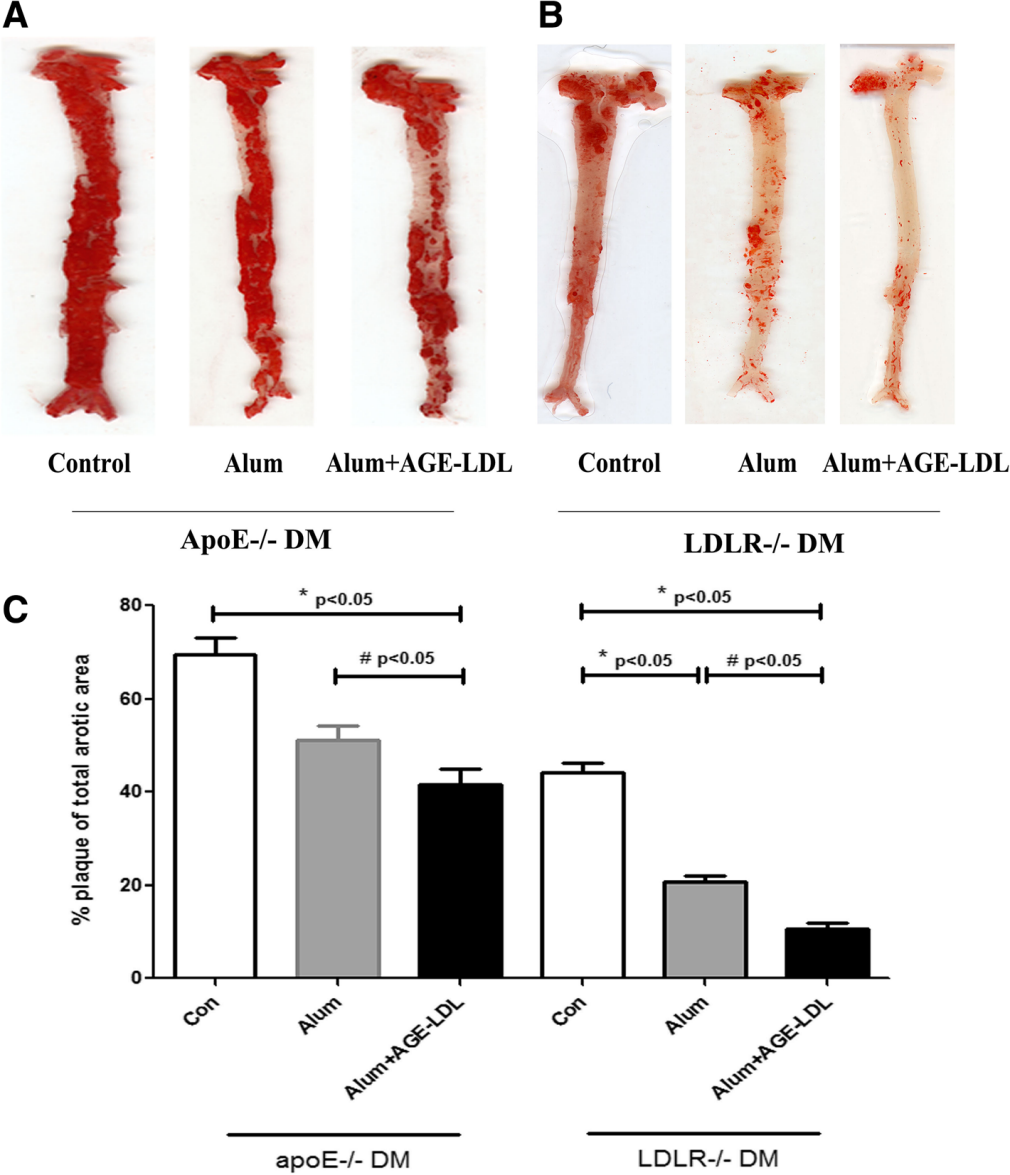
**Immunization with AGE-LDL using Alum as adjuvant reduced atherosclerotic lesion burden of diabetic apoE −/− and LDLR-/ mice. A** and **B**, Representative photomicrographs showing oil red O stained en face preparations of aortas (n = 10 in each group). **C**. Percentage of plaque area of total aortic area of diabetic apoE−/− and LDLR−/− mice treated with Alum adjuvant (bar with stripes), Alum + AGE-LDL immunization (black bar), and PBS treated controls (white bar). Mean ± SD values are shown. *indicates p < 0.05 vs. control, **indicates p < 0.01 vs. control, #indicates p < 0.05 vs. Alum adjuvant.

**Figure 2:**
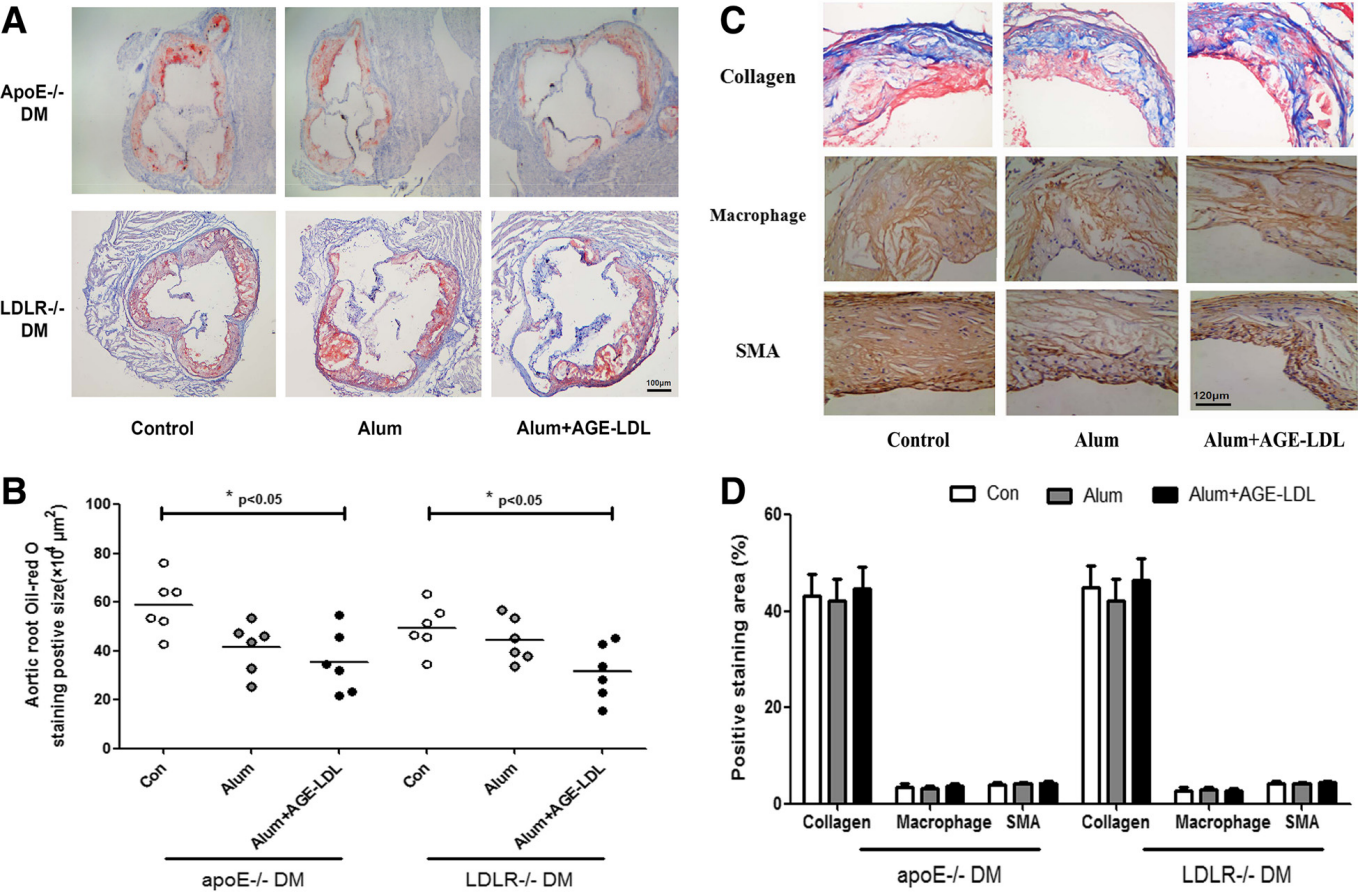
**Immunization with AGE-LDL and Alum adjuvant did not significantly change atherosclerotic lesion composition in the aortic root of diabetic apoE −/− and LDLR−/− mice. A** and **C**, Representative photomicrographs showing Oil-red O (scale bar = 100 μm) and collagen (Masson), macrophage, and smooth muscle cell (SMA) stained aortic root sections (scale bar = 120 μm) from each group (n = 6 per group). **B**. Oil-red O stained area in the aortic root: white circles represent animals from the PBS-treated control group, black squares represent animals from the Alum adjuvant group, and white squares represent animals from the AGE-LDL + Alum treated group. *indicates p < 0.05 vs. control. **D**, Quantification of collagen, macrophages and smooth muscle cells from the 3 groups are shown: white bars represent animals from the control group, bars with stripes represent animals from the Alum adjuvant group, and black bars represent animals from AGE-LDL + Alum treated group.

Immunization with AGE-LDL using Alum as adjuvant resulted in a significant reduction in atherosclerotic lesion burden compared to mice immunized with PBS control (76% in LDLR null diabetes mellitus(DM) mice, p < 0.01; and 43% in apoE null DM mice, p < 0.01) or Alum alone (50% in LDLR null DM mice, p < 0.05; and 20% in apoE null DM mice, p < 0.05) (Figure 1). AGE-LDL immunization also reduced lesion area in the aortic root (42% in apoE null DM mice, p < 0.05; 40% in LDLR null DM mice, p < 0.05) compared with PBS controls (Figure 2A-B). Atherosclerotic lesions were only significantly attenuated by administration of Alum adjuvant compared with PBS controls in LDLR−/− DM mice but not in apoE−/− DM mice, which was consistent with previously reported anti-atherosclerotic effects of Alum therapy [36] and a discrepancy in vaccination response between the two hypercholesterolemic mouse models. However, quantitative immunohistochemical analysis of markers for macrophages (MOMA-2), smooth muscle cells (alpha-smooth muscle actin) or collagen (Masson) showed that the immunization strategy did not significantly alter the composition of the lesions (Figure 2C-D).

### Immunization with AGE-LDL increases plasma HDL levels

Immunization with AGE-LDL using Alum adjuvant increased serum HDL levels both in apoE and LDLR DM mice (151.97 ± 25.08 mg/dl and 153.91 ± 21.14 mg/dl) compared with PBS controls (113.30 ± 15.47 mg/dl and 127.11 ± 21.33 mg/dl) (P < 0.05), without significant effects on body weight, serum cholesterol, or non-fasting glucose levels. Although HbA1c levels were not significantly different among groups, numerically lower HbA1c level was found in both mouse models (p = 0.068 in apoE DM mice and p = 0.073 in LDLR DM mice for% data, while p = 0.059 in apoE ones and p = 0.074 in LDLR ones for mmol/L data). The data suggest that part of the atheroprotective effects of vaccination with AGE-LDL could be derived from improved metabolic control (Table 2).

**Table 2:** **Weight, cholesterol, average plasma non-fasting glucose and HbA1c levels**

	**Group**	**Weight (g)**	**Cholesterol**	**HDL-C**	**LDL-C**	**Average non-fast glucose**	**HbA1c**	
			**(mg/dl)**	**(mg/dl)**	**(mg/dl)**	**(mg/dl)**	**(%)**	**(mmol/L)**
apoE −/− DM	Control (n = 12)	28.11±1.40	1082.46±137.40	113.30±15.47	767.10±45.40	547.99±36.85	9.10±0.45	76.00 ± 4.93
	Alum (n = 12)	28.64±2.53	1072.06±144.53	136.12±23.20	752.57±55.88	557.63±29.55	8.76±0.40	72.13 ± 4.29
	Alum + AGE-LDL (n = 12)	28.17±2.43	1084.85±138.43	151.97±25.08*	739.37±44.92	548.53±44.33	8.58±0.43	70.25 ± 4.56
LDLR −/− DM	Control (n = 10)	27.11±1.35	1077.65±115.22	127.11±21.33	781.86±44.43	531.51±42.40	8.72±0.16	71.88 ± 1.73
	Alum (n = 10)	28.03±2.11	1085.10±154.39	138.44±20.42	778.58±33.78	548.52±51.53	8.67±0.17	71.38 ± 1.85
	Alum + AGE-LDL (n = 10)	28.21±1.03	1090.24±143.53	153.91±21.14*	768.55±44.97	536.35±42.65	8.52±0.13	69.88 ± 1.55
	P value	n.s.	n.s.		n.s.	n.s.	n.s.	n.s.

Mean values and standard deviations are shown. *p < 0.05 vs. PBS control.

### Immunization with AGE-LDL induces systemic humoral and cellular immune responses

**Figure 3:**
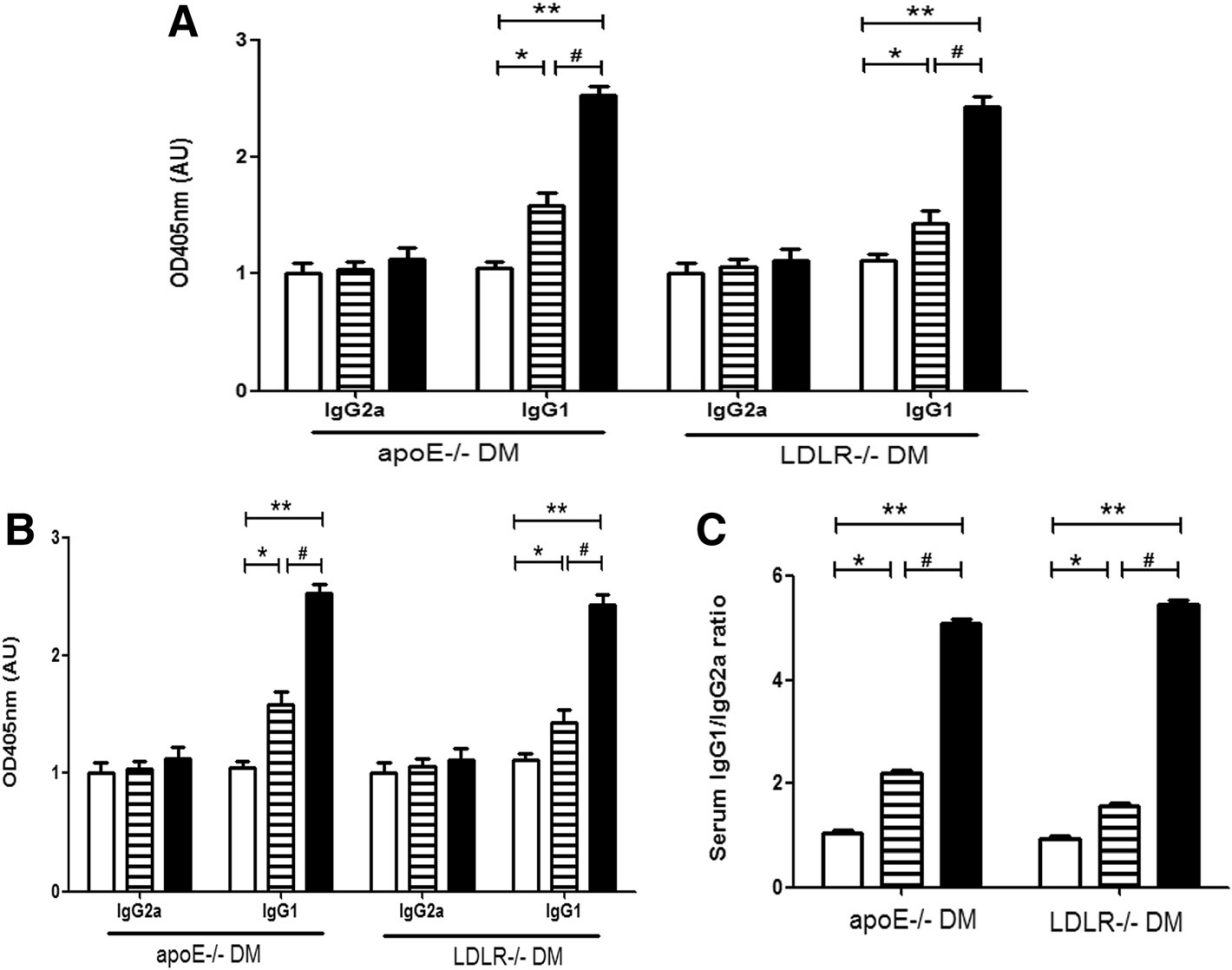
**Serum titers of IgG, IgM, IgG1 and IgG2a antibodies. A**. ELISA analysis of titers of IgG, IgM, and specific anti-AGE-LDL IgG and IgM antibodies in sera from diabetic apoE−/− and LDLR−/− mice (n = 10 per group) treated with Alum adjuvant (bar with stripes), AGE-LDL + Alum adjuvant (black bar) or PBS controls (white bar). **B**. ELISA analysis of specific IgG1 and IgG2a antibodies against AGE-LDL in sera from diabetic apoE−/− and LDLR−/− mice treated with Alum adjuvant, AGE-LDL + Alum adjuvant or PBS controls. **C** shows the ratio of serum IgG1/IgG2a antibodies among three groups. *indicates p < 0.05 vs. control, **indicates p < 0.01 vs. control, and #indicates p < 0.05 vs. Alum.

Immunization with AGE-LDL using Alum as adjuvant induced significantly elevated titers of IgG and IgM antibodies against AGE-LDL in both diabetic apoE −/− and LDLR −/− mice (Figure 3A). Total IgG and IgM levels were not influenced by either treatment. Immunization with Alum alone did not induce AGE-LDL-specific IgM and IgG titers implying that the humoral immune responses were specific to the antigen AGE-LDL and not associated with adjuvant. Further analysis showed that the increase of IgG level was mostly derived from that of IgG1, associated with a Th2 response, which was significantly increased in serum from AGE-LDL immunized mice compared with serum from control or Alum adjuvant treated groups (p < 0.05, Figure 3B). Modestly increased anti-AGE-LDL IgG level was observed in Alum adjuvant-immunized mice, which might be associated with antigen capture as previously reported [36]. The IgG1/IgG2a ratio of anti-AGE-LDL antibodies was significantly increased in both models receiving AGE-LDL vaccination compared with Alum and PBS control recipients (p < 0.05 and p < 0.01 respectively) (Figure 3C), with increased Th2 levels without significant change of Th1.

**Figure 4:**
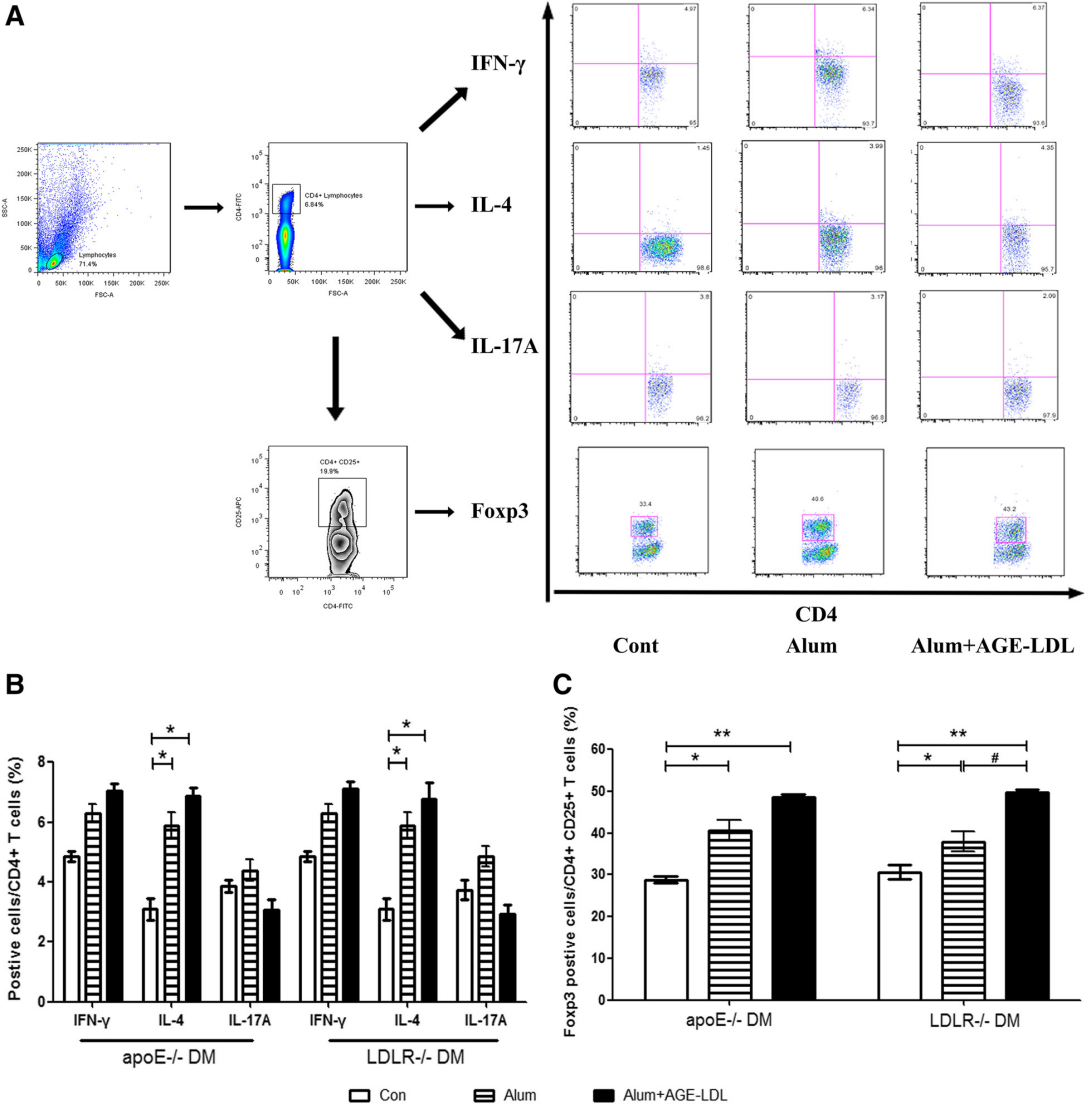
**T cell subsets in spleen after immunization. A**. Flow cytometry analysis strategies of T cell subsets in spleen, **B** and **C**. Levels of T cell subset are shown as percentages of cytokine producing cells (Interferon-γ, IL-17 and IL-4) per CD4+ T cells, and FoxP3 positive cells per CD4+ CD25+ T cells stimulated with AGE-LDL for each of the three groups (white bars = controls, bars with stripes = Alum adjuvant and black bars = AGE-LDL + Alum adjuvant, n = 10 per group). *indicates p < 0.05 vs. control, **indicates p < 0.01 vs. control, and #indicates p < 0.05 vs. Alum.

Data from flow cytometry analysis showed that combined vaccination with Alum and AGE-LDL significantly increased the proportion of Th2 and Treg CD4^+^ T cells subsets in spleen, characterized by intracellular staining for IL-4 and FoxP3, while no significant differences were shown in Th1 and Th17 cells compared with those stimulated with AGE-LDL from control and Alum adjuvant treated groups (Figure 4A-C), although numerically lower Th17 for apoE or LDLR null mice was shown in AGE-LDL immunized vs. Alum alone treated mice (p = 0.08 in apoE DM mice and p = 0.075 in LDLR DM mice).

### Immunization with AGE-LDL increases splenic FoxP3 and IL-10 mRNA levels and cytokine secretion

**Figure 5:**
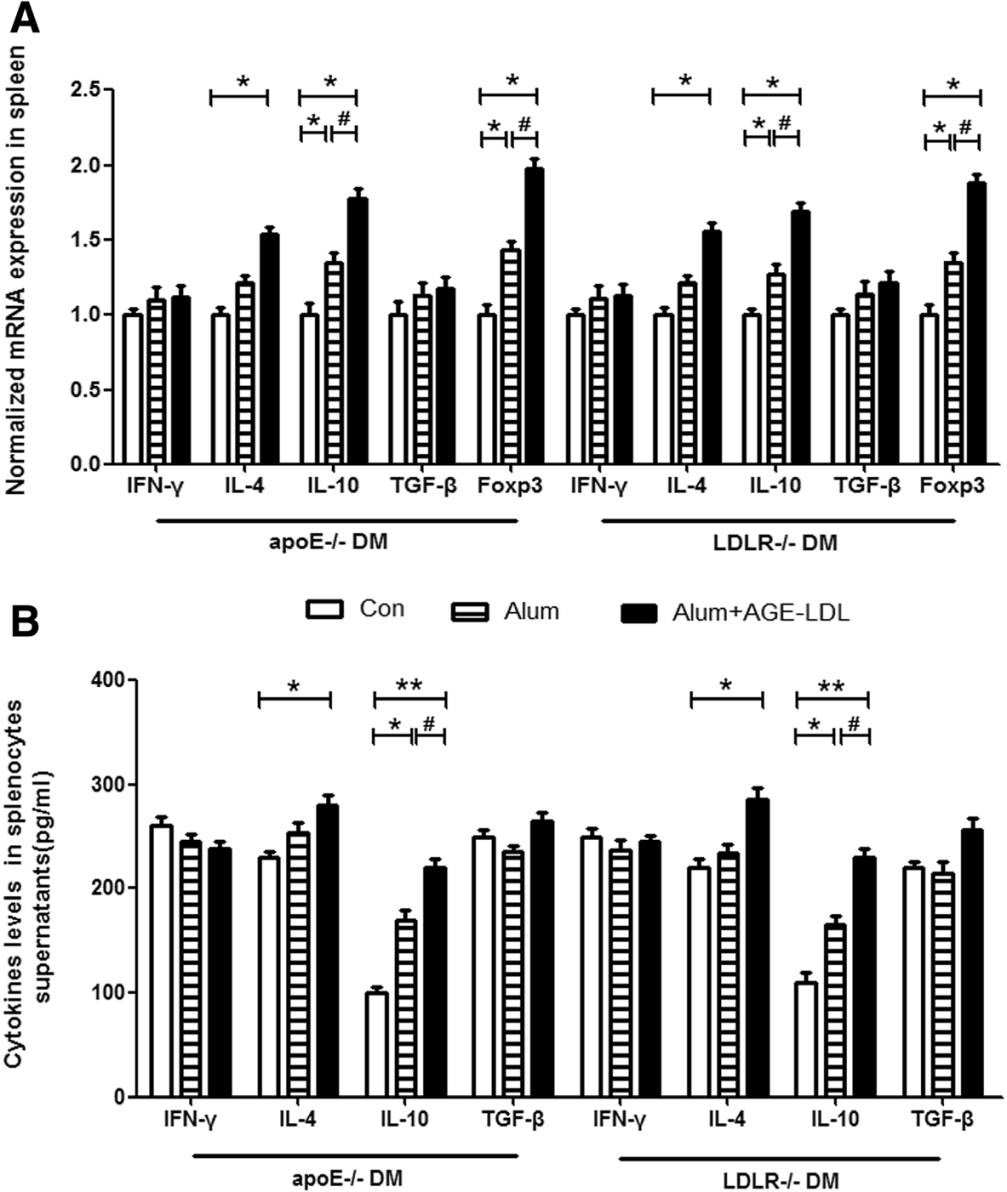
**Immunization with AGE-LDL increased IL-4, FoxP3 and IL-10 levels in spleens from diabetic mice. (A)** mRNA transcript ratios based on GAPDH expression are shown for each gene of interest for all 3 groups (n = 10 per group). **(B)** Secretion patterns of cytokines determined in supernatants derived from splenocytes stimulated with AGE-LDL among groups were analyzed with milliplex assay. White bars = controls, bars with stripes = Alum adjuvant and black bars = AGE-LDL + Alum adjuvant. *indicates p < 0.05 vs. control, **indicates p < 0.01 vs. control, and #indicates p < 0.05 vs. Alum.

Real-time PCR analysis of splenocytes from apoE and LDLR null mice immunized with AGE-LDL showed significant increases in FoxP3 and IL-10 mRNA levels compared to both PBS and Alum treated mouse groups (Figure 5A). Alum alone also induced an increase in IL-10 and Foxp3 mRNA levels. Protein level of IL-10 was significantly elevated in AGE-LDL compared with Alum alone treated mice as well as in Alum treated mice versus control group (Figure 5B), which was consistent with real-time PCR data. In addition, there was numerically higher transforming growth factor-beta (TGF-**β**) (p = 0.23) mRNA level in Alum treated mice compared with PBS controls (Figure 5A).

## Discussion

The present study provides a novel strategy, namely subcutaneous administration of AGE-LDL using Alum as adjuvant, to induce protective immunity against atherosclerosis in two complex animal models with diabetes and atherosclerosis. The anti-atherogenic effect could be attributed to increased levels of HDL, antigen-specific Th2 antibodies and IL-10, and expansion of antigen-specific CD4^+^ Treg cells.

### Mechanisms behind the vaccination strategy

Only diabetic atherosclerotic model mice were chosen in the present study to evaluate the efficacy of the vaccination strategy, which was based on previous studies showing that increased level of AGEs was found mainly in patients with diabetes and end-stage renal diseases [37-39] and contributed to accelerated atherosclerosis.

In addition to the above patient populations, data from Japan Assessment of Pitavastatin and Atorvastatin in Acute Coronary Syndrome (JAPAN-ACS) trial showed that high baseline AGEs levels were also associated with plaque progression among patients with AS independently of DM [40]. Meanwhile AGEs was shown to be related to long-term glucose control and other oxidative stresses in acute coronary syndrome [40] and in the development of coronary artery calcification in individuals with type 1 DM [41].

Thus it is likely that immunization with AGE-LDL would have its most prominent effect in diabetic mice. However, because glycation of LDL, and especially of small-dense LDL, also has been found in patients without diabetes [42], and there is some cross-reactivity between ox-LDL and AGE-LDL antibodies [22], it cannot be excluded that immunization with AGE-LDL also could reduce atherosclerosis, albeit to a lesser extent, in animal models without diabetes. However, this needs to be further explored.

Immunization with different antigens inhibits atherosclerosis progression in atherosclerosis-prone animal models [6,9,32,35,43], however, there are scarce data on efficacy of such immunization strategies in diabetic animal models with atherosclerotic disease. Research in this field is highly important because diabetics account for a very large proportion of patients requiring cardiovascular therapy; incidence of diabetes continues to rapidly increase in both developed and developing countries [44,45]; and diabetes is associated with increased risk of cardiovascular disease.

As the main core etiological factors, data from recent studies confirmed that blockade of the positive feedback loop between AGE-receptor for advanced glycation end products (RAGE) axis by dipeptidyl peptidase-4 inhibitors might be a novel therapeutic target for vascular injury in diabetes [46], and furthermore exendin-4 could exert cardioprotective effect against diabetic cardiomyopathy, which may be associated with the inhibition of RAGE expression [47]. Except for AGE-RAGE, heparanase is another target gene of the diabetic nephropathy mediators albumin and AGE [48], which was proved by experimental evidence to play a key role in AGEs-induced macrophage migration associated with inflammation in diabetic vascular complication [49]. All the above might be the potential mechanism of our vaccination strategies.

The present findings not only confirm the efficacy AGE-LDL immunization in inhibiting atherosclerosis progression, but also show some interesting and unexpected findings: 1) Increased serum HDL and numerically lower HbA1c levels in mice receiving AGE-LDL vaccination, although glucose levels did not differ significantly among groups; improved metabolic control therefore might be one of the protective effects of vaccination in accordance with a previous report showing anti-diabetic effects of all-trans retinoic acid secondary to increasing Treg levels [50,51]. 2) Although apoE−/− and LDLR−/− mice are used as atherosclerosis-prone mouse models, mechanism of atherosclerosis progression differs between the two models. The present study showed that vaccination with Alum significantly decreased plaque burden in subvalvular aorta only in LDLR null DM mice but not in apoE null DM mice, which might reflect the intrinsic difference of genetic background between the two mouse models. A previous study showed that targeted deletion of Group V secretory phospholipase A2 could significantly alter atherosclerotic lesion area in LDLR−/− mice but not apoE−/− mice, which was attributed to different composition and oxidative status of LDL between such strains [52], and might also lead to the discrepancy of atherosclerosis burden observed in our study. However, because immunization with AGE-LDL and Alum as adjuvant has similar effects and mechanisms in both models, the immunization strategy based on AGE-LDL appears as a promising option for vaccine development.

### Current strategies against atherosclerosis

Several immunization strategies against atherosclerosis have been developed, and different techniques and targets have been chosen to determine their protective effect in mice and rabbits [53,54]. Among these antigens, apolipoprotein B100 (ApoB100, CVX-210-H) and the cholesterolester transferase protein (CETP) were mostly chosen and shown to be well-tolerated among patients [55]. However, there were several limitations to their ability to induce protective immunity against atherosclerosis, including insufficient immunogenicity; further formulations with different carriers or adjuvants need to be tested. In our study, Alum was chosen to enhance the immunological effects of AGE-LDL because it 1) is an adjuvant that has be used in humans for many years; and 2) has been proven to mediate beneficial effects against atherosclerosis by capturing oxidized LDL and activating Tregs [36].

## Conclusion

The present study showed that combined treatment with human AGE-LDL and Alum adjuvant induces effective immunity against atherosclerotic progression in diabetic animal models with atherosclerosis, possibly mediated by activation of protective autoantibodies production and Tregs, as indicated by increased AGE-LDL specific IgM and IgG titers and Foxp3 and IL-10 expression, respectively. Study results also indicate that such treatment might be useful to improve metabolic status, as shown by increased HDL and numerically lower HbA1c levels, although the mechanism for the metabolic modulation remains unknown. Vaccination with AGE-LDL potentially offers a novel approach for treatment of atherosclerosis in patients with diabetes; however, results of the present study need to be interpreted with due caution and further research is warranted on safety and efficacy.

## Additional file

Additional file 1: Figure S1. Detailed experimental protocol. The experimental design of studies utilized three groups of female apoE and LDLr-/- mice at 6 weeks of age. Mice were fed ad libitum a high-cholesterol diet and made diabetic with intraperitoneal injections of streptozotocin for 7 days; and monitored for blood glucose and HbA1c once weekly until euthanized. At 9 weeks of age the mice were injected subcutaneously (first immunization) in the dorsal area between scapulas, followed by a booster at 11, 13 and 15 weeks of age. Aluminum hydroxide (Alum) (Biosecter, Denmark) was used as adjuvant and mixed with AGE-LDL (25 μg/dose) with 1:1 ratio in volume. Alum alone or citrate buffer was used as controls. All mice were euthanized at 24 weeks of age to analyze atherosclerosis burden and immunity response.

## Abbreviations

AGE-LDL: Advanced glycation end product -modified low-density lipoprotein
AGEs: Advanced glycation end products
Alum: Aluminum hydroxide
APES: 3-aminopropyltriethoxysilane
AS: Atherosclerosis
CVD: Cardiovascular disease
CETP: Cholesterolester transferase protein
DM: Diabetes mellitus
FMO: Fluorescence minus one
HDL: High density lipoprotein
HDL-C: High density lipoprotein cholesterol
HMF: 5-hydroxymethylfurfural
JAPAN-ACS: Japan Assessment of Pitavastatin and Atorvastatin in Acute Coronary Syndrome
LDL-C: Low density lipoprotein cholesterol
LDLR: LDL receptor
nLDL: Native LDL
MDA: Malondialdehyde
ox-LDL: Oxidized low density lipoprotein
PBS: Phosphate-buffered saline
PMA: Phorbol 12-myristate 13-acetate
RAGE: Receptor for advanced glycation end products
TBA: Thiobarbituric assay
TBARS: Thiobarbituric acid reactive substances
TGF-β: Transforming growth factor-beta
TNBSA: Trinitrobenzene sulphonic acid assay
