# Supplementary figures and images for: Immunization with advanced glycation end products modified low density lipoprotein inhibits atherosclerosis progression in diabetic apoE and LDLR null mice
Source: Cardiovasc Diabetol. 2014 Nov 13;13:151. doi: 10.1186/s12933-014-0151-6 (PMC4234834; doi:10.1186/s12933-014-0151-6)

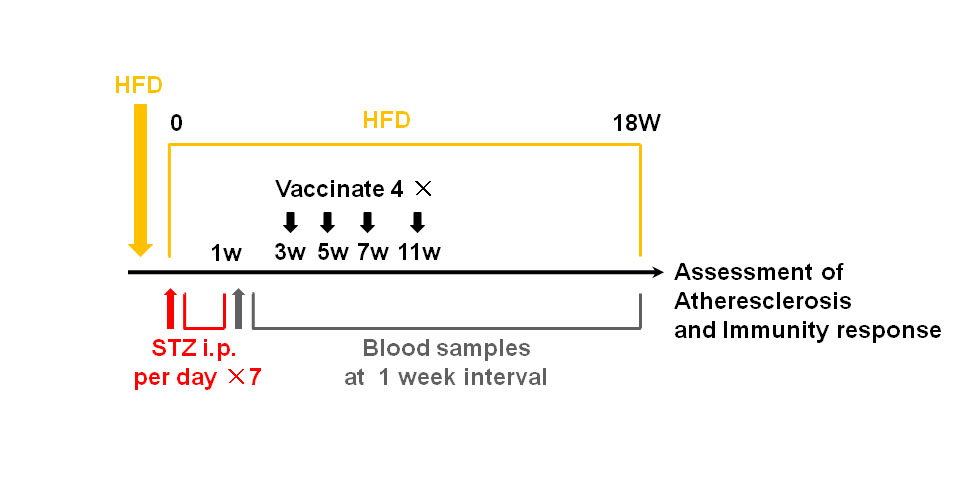

Supplement: Additional file 1: Figure S1. — Detailed experimental protocol. The experimental design of studies utilized three groups of female apoE and LDLr-/- mice at 6 weeks of age. Mice were fed ad libitum a high-cholesterol diet and made diabetic with intraperitoneal injections of streptozotocin for 7 days; and monitored for blood glucose and HbA1c once weekly until euthanized. At 9 weeks of age the mice were injected subcutaneously (first immunization) in the dorsal area between scapulas, followed by a booster at 11, 13 and 15 weeks of age. Aluminum hydroxide (Alum) (Biosecter, Denmark) was used as adjuvant and mixed with AGE-LDL (25 μg/dose) with 1:1 ratio in volume. Alum alone or citrate buffer was used as controls. All mice were euthanized at 24 weeks of age to analyze atherosclerosis burden and immunity response. [file 12933_2014_151_MOESM1_ESM.jpeg]
